# Annotations

**Published:** 1899-10-07

**Authors:** 


### Annotations.

### Chloroform Administration.

With the opening session just before us, and with the prospect of that renewed activity in the operating theatres of all our hospitals which usually characterises the October term, is it too much to ask that the greatest possible care shall be exercised in the administration of anæsthetics, more especially by junior officials, and that when it is found desirable to give chloroform this drug shall be administered in such a manner as to conform to the principles generally accepted in regard to other drugs, namely, that it shall be given in measured doses? It may be admitted at once that there are certain difficulties in so doing from the very nature of the drug, but that should not stand in the way of so desirable a consummation. We do not wish to enter upon questions of dosage, of extent of dilution by air, or of regularity of administration; all we ask is that the anæsthetist, in dealing with chloroform, shall know what he is doing. If he thinks it well to give heavy doses, we will not now argue the question whether such a method is right or wrong, but we do urge that he shall know with some approach to exactitude how much he is giving, breath by breath, throughout the whole time of the operation. This is almost impossible by what is called the open method—quite impossible as this is ordinarily carried out—but there are various forms of apparatus by which it can be done. Some of these are more easy to manipulate than others, but the principle is the same in all, namely, that the quantity of the drug given shall be under the immediate control of the administrator, and that it shall be capable of regulation breath by breath. And this should be looked upon as essential.

### From Street Noises to Church Bells.

Among the various mercies which are promised us in the year 1900 is the coming into force of the London Government Act, which contains a provision of much interest to all who possess "nerves," for among the powers granted by it to the new municipality is that of making bye-laws for regulating and suppressing street noises. What possibilities rise before the mind at the thought of a quiet London! We need not go over the subject of the injury done to the nervous system of those who are compelled to live amid the roar and racket of our great cities, but we would just point out that, when our authorities have managed once to put a check upon some of the more prominent and irritating noises of the street, other noises, not exactly of the street, which at present are to a large extent drowned amid the ever-present tumult, will then begin to attract attention, and among them will be church bells. The writer of an article which recently appeared in the *Times* regrets the neglect which has fallen upon many belfries and the decadence of "scientific ringing" which has accompanied it. "The restorers of St. Saviour's, Southwark — hereafter, perhaps, to be the cathedral of South London—have," he says, "declined even to consider the rehanging of its grand peal of twelve, now lying voiceless and discredited." We are also told that within the present generation there has been, and there still is going on, an active movement for reform both in belfries and among ringers; that now a revival of the art of change ringing is taking place; that diocesan or county societies for its encouragement have been formed all over the country; and lastly, that the art now possesses even its own literary organ. Alas! alas! For, indeed, to those who live close to them, bells are an unmitigated nuisance, annoying enough even to the healthy, but actual torture to the sick. The distant sound of "bells at evening pealing" is to some of us full of pleasant associations, and to those who listen at a distance is a sweet sound in itself; but the hammering crash of a noisy peal, bellowing out relentlessly its never-ending changes in the midst of town or village, is a nuisance which no community should put up with. We are told that a "Central Council of Church Bellringers" meets annually in London, and that it is representative of some thirty thousand ringers; and as we are also assured that well-ascertained means exist for diminishing without entirely silencing the sound of bells in close proximity to houses, we would urge this body to use its influence to enforce the application of these means to all town churches. Certain it is that if ever serious action is taken to suppress the ordinary trade noises of the street the movement will not stop there, but will demand some mitigation of steam whistles and church bells.

### Infants' Food—Tea, Cheese, and Beer.

According to the *Globe*, the Newington Vestry has issued a warning to the mothers of the district that cheese, beer, and tea are bad for babies. But we doubt if the warning will do much good. The mothers of Newington know quite well that the articles specified are unwholesome for children. We cannot plead for them, when they drive their children into convulsions that they have sinned in ignorance. They do not, indeed, fully realise the consequences of their ill-feeding, but that is because they have never given any thought to them. If questioned they would admit that they knew, or had at least been told, that milk, or some kind of milk food, was the proper sustenance for infants, but they would probably add that "babies like a bit of what's going." The truth is, their sins are the result of laziness and want of thought. Infants' food requires careful preparation, not always easy to manage over the smoky fire in their pokey kitchen, where milk sours with alarming rapidity. But cheese and beer require no preparation, and there is sure to be a pot of tea stewing by the fire for their own delectation, and a taste of these dainties, if it does not nourish the baby, tends at least to keep it quiet. That is all they care for. And anyone who knows how burdensome a fretful child may be to a tired and overworked woman will sympathise with them and wish to see them get a little rest. But they go the wrong way about it. If they reflected they might know. But that class do not reflect. They go on doing what seems at the moment to be the easiest thing, and in all probability they will go on doing so in spite of all the warnings the vestry can issue. But the fact that it has seemed necessary to issue such warning shows that in England, as in Napoleonic France, we require "a generation of mothers"—with some notion of the duties of motherhood.

